# An exploration of the knowledge and attitudes towards breastfeeding among a sample of Chinese mothers in Ireland

**DOI:** 10.1186/1471-2458-10-722

**Published:** 2010-11-23

**Authors:** Qianling Zhou, Katherine M Younger, John M Kearney

**Affiliations:** 1 School of Biological Sciences, Dublin Institute of Technology, Kevin Street, Dublin 8, Republic of Ireland

## Abstract

**Background:**

Psychological factors are important in influencing breastfeeding practices. This retrospective study explored knowledge and attitudes related to breastfeeding of Chinese mothers living in Ireland.

**Methods:**

A cross-sectional self-administrated survey written in Chinese was distributed to a convenience sample of 322 immigrant Chinese mothers mainly *via *Chinese supermarkets and Chinese language schools in Dublin, with the involvement of the snowball method to increase sample size. Maternal breastfeeding knowledge and attitudes were described, their associations with socio-demographic variables were explored by Chi-square analysis, and their independent associations with breastfeeding behaviours were estimated by binary logistic regression analyses.

**Results:**

In spite of considerable awareness of the advantages of breastfeeding (mean score = 4.03 ± 0.73), some misconceptions (*e*.*g*. 'mother should stop breastfeeding if she catches a cold') and negative attitudes (*e*.*g*. breastfeeding inconvenient, embarrassing, and adverse to mothers' figure) existed, especially among the less educated mothers. Cultural beliefs concerning the traditional Chinese postpartum diet were prevalent, particularly among those who had lived in Ireland for a shorter duration (P = 0.004). Psychological parameters had strong independent associations with breastfeeding practices in this study. Those who had lower awareness score (OR = 2.98, 95% CI: 1.87-4.73), more misconceptions and negative attitudes (P < 0.05), and weaker cultural beliefs (P < 0.05) were less likely to breastfeed.

**Conclusions:**

Findings highlight a need to focus resources and education on correcting the misconceptions identified and reversing the negative attitudes towards breastfeeding among Chinese mothers in Ireland, in particular those with primary/secondary level of education. Mothers' cultural beliefs should also be acknowledged and understood by healthcare providers.

## Background

The benefits of breastfeeding have been well recognized [1], and education about and promotion of breastfeeding have become a public health focus worldwide. Breastfeeding practices and attitudes are influenced by demographic, biophysical, social, cultural and psychological factors [2-5]. Migration to another country may induce to some changes in maternal infant feeding attitudes and practices. The associations between breastfeeding beliefs [6] as well as behaviours [7] and the degree of maternal acculturation have been identified.

In spite of high breastfeeding rates in China and a strong Chinese breastfeeding culture [8,9], such prevalence is not always the case among Chinese immigrants in Western countries. Studies have found that immigrant Chinese mothers in Europe and North America seldom nursed their children [10,11]. A cross-sectional study in Toronto revealed a breastfeeding rate of 18% at six months and the complementary food choices of the Chinese mothers reflected both Western and Eastern influences. The authors suggested that the abandonment of breastfeeding was due to maternal cultural transition [12]. Goel *et al*. [10] in Scotland attributed the lack of breastfeeding intention to mothers' wrong information or misconceptions about British infant feeding practices, their language problem and their infrequent attendance of child welfare clinics. Studies in Australia reported the common perceptions of inadequate breast milk and inconvenience of breastfeeding [13,14]. Li *et al*. [15] in Perth, Australia discovered the maintenance of some traditional practices among Chinese mothers who gave birth in Australia, *e*.*g*. using traditional methods to boost breast milk production. This survey also detailed Chinese immigrant mothers' knowledge and attitudes about breastfeeding [16].

Ireland, a country with one of the world's lowest levels of breastfeeding [17], has in the last decade become a multicultural society comprised of immigrants from diverse ethnic backgrounds [18]. Several campaigns have been launched to improve breastfeeding in Ireland. For example, the five-year Strategic Action Plan for Breastfeeding advised better hospital and community breastfeeding services for mothers, in order to raise the breastfeeding initiation rate by 2% points per year [19]. A cohort study (n = 450) in a large maternity hospital in Dublin conducted between 2004 and 2006 has demonstrated that breastfeeding initiation rate of Irish nationals (47%) was significantly lower that of non-nationals (79.6%) [20]. Similar findings have also been reported in the most recent infant feeding survey in Ireland [21]. Owing to the small size of each non-national group, breastfeeding rates of each group have not been detailed in either of these studies, however, both studies suggested a need for health professionals to understand cultural and attitudinal differences in breastfeeding. However, the attitudinal parameter, a strong predictor of infant feeding practices [22,23], has scarcely been examined in Ireland [24]. Hence, this study was undertaken to explore the knowledge and attitudes towards breastfeeding of the Chinese, a major ethnic population in Ireland [18], and determine the influence of socio-demographic factors on maternal knowledge and attitudes, in an attempt to facilitate the appropriate promotion and health support of breastfeeding to the immigrant Chinese mothers in Ireland, and to contribute to the overall increase of breastfeeding rates in Ireland.

## Methods

### The sample

Subject inclusion criteria were: women who were born in China (including Hong Kong and Macau), who had given birth to at least one child, and had been in Ireland for at least 6 months.

### Data collection

Ethical approval was obtained from the Research Ethics Committee of the Dublin Institute of Technology. The study was announced in a Chinese local newspaper and an internet forum which are frequently visited by the Chinese in Ireland, as well as some Chinese local communities. Recruitment was between September and December 2008. Potential participants were approached in Chinese supermarkets, Chinese Language Schools, church organizations and a few Chinese restaurants in Dublin urban and suburban areas. The purpose and demands of the study were explained (in Chinese), confidentiality and anonymity were assured, and the questionnaire was distributed to each participant, together with instructions for completion and a stamped addressed envelope. A 'snowball' technique was used to increase sample size, *i*.*e*. participants were requested to spread word of this study and distribute the questionnaires to their friends or relatives. A total number of 343 questionnaires were collected by post. Where necessary, respondents were re-contacted by a follow-up telephone call to clarify or fill in missing information. Participants who were born outside China as well as those whose child(ren) were born neither in China nor in Ireland were further excluded. A final total of 322 questionnaires were used in the data analysis.

### The questionnaire

The cross-sectional questionnaire was devised on the basis of an extensive literature review of migration studies [13,14,16,25,26]. This paper mainly used questions in the final section, which were specifically designed to examine the maternal knowledge and explore the attitudes of breastfeeding. Breastfeeding knowledge was assessed by four questions (S1-S4) related to the advantages of breastfeeding, as well as some other questions on the fundamental understanding of breastfeeding [16,25]. Some breastfeeding attitude questions were adopted from the Iowa Infant Feeding Attitude Scale (IIFAS) [26], which has been used in several different populations [22,26-28]. Additional attitude questions were related to the Chinese breastfeeding culture and context [13,14,16,29,30] and mother's views on the Irish breastfeeding environment. A 5-point Likert scale was used to test the maternal awareness of the advantage of breastfeeding to the babies (S1-S4). The higher the score, the greater the level of awareness. As for the other knowledge items and attitudes, a 4-point Likert scale was used. Socio-demographic information (mother's current age, marital status, birthplace, education level, duration of staying in Ireland, delivery location, couple's occupations and family income), as well as questions on whether mother had breastfed one or more children, and whether the mother herself had been breastfed as a baby were also asked.

The questionnaire was reviewed for content validity and cultural appropriateness by two breastfeeding specialists and a Chinese medical doctor. Members of the panel checked and made comments on the questionnaire individually. Subsequent adjustments of the questionnaire were made accordingly and then approved by all members in the panel. Afterwards, the questionnaire was translated into Chinese and blind back translated for the accuracy of the translation [31], and the Chinese version was also checked for language adequacy. The final questionnaire was then pilot tested on 20 Chinese mothers recruited in Chinese supermarkets in Ireland to assess clarity and redundancy.

### Data analyses

Data analyses were conducted with SPSS (version 15). Frequencies and descriptive summary statistics were performed to describe the sample. Cross-tabulations and Pearson's Chi-square tests was performed to assess the association of socio-demographic variables with knowledge and attitudes towards breastfeeding. The mean awareness score was calculated by summing all awareness scale items and dividing by the total number of items. The internal consistency (Cronbach's alpha) of the awareness scale was 0.84, indicating acceptable reliability. One-way analysis of variance (ANOVA) at the 95% significance level was conducted to compare the difference in mean awareness scores across socio-demographic status. Where statistically significant effects were encountered (P < 0.05), comparisons of means were made using Scheffe's *post hoc *multiple comparisons test. Binary logistic regression analyses were performed to assess the independent associations between breastfeeding knowledge and attitudes, and breastfeeding behaviours after controlling for socio-demographic confounders. Attitude/knowledge items were accessed individually, *i*.*e*. in each analysis, one attitude/knowledge variable together with all the socio-demographic variables (including maternal current age, maternal birthplace, mother's duration in Ireland, education level, whether or not mother herself had been breastfed when she was a baby, and husband/partner's occupation) were entered into the model in a forced entry fashion. Odds ratio (OR) and 95% confidence intervals (CI) were reported. The level of 5% significance was used throughout the statistical analyses.

In this paper, breastfeeding is taken to mean 'any breastfeeding', *i*.*e*. the child has received breast milk with or without other drink, formula or other infant food [9]. 'Breast-feeder' refers to Chinese mothers who have breastfed at least one child; and 'non-breast-feeder' as Chinese mothers who have never breastfed any of their children.

## Results

### Sample characteristics

This study included 322 Chinese mothers living in Ireland (mainly Dublin and its suburban areas) at the time of the study, 77.2% had their child(ren) in Ireland, 15.0% delivered in China, and the rest 7.8% gave birth in both China and Ireland. Mothers were 22 to 54 years old (mean age 33.2 years), staying in Ireland from 0.5 to 29.1 years (mean duration 7.5 years). The majority were married, housewives, born in the northern part of mainland China, had a third level education and had a family income of 15,000-30,000 euro annually. Most mothers had breastfed at least one child (breast-feeder) and had been breastfed as a baby. Husband/partner of respondents was mainly a non-professional worker (Table 1).

**Table 1:** Characteristics of the sample (n = 322)

Characteristic	No. ^†^	(%)
*Current age*		
20-29	107	(34.0)
30-39	154	(48.9)
> 40	54	(17.1)
*Marital status*		
Married	275	(85.4)
Single/Divorced/Widow	47	(14.6)
*Education level*		
Primary/Secondary	156	(48.4)
Tertiary	166	(51.6)
*Current occupation*		
Self-employed/Professional work	79	(24.9)
Non-professional work	107	(33.8)
Housewife	131	(41.3)
*Husband/partner occupation*		
Self-employed/Professional work	109	(34.8)
Non-professional work	195	(62.3)
Unemployed	9	(2.9)
*Family income last year (euro, before tax)*		
< 15,000	49	(15.7)
15,000-30,000	163	(52.1)
30,000-50,000	59	(18.8)
> 50,000	42	(13.4)
*Mother's birth place*^§^		
Mainland China (North)	164	(51.3)
Mainland China (South)	109	(34.1)
Hong Kong	47	(14.7)
*Duration in Ireland (years)*		
< = 5	99	(30.7)
> 5-10	179	(55.6)
> 10	44	(13.7)
*Has mother herself been breastfed when she was a baby?*		
Yes	276	(85.7)
No	31	(9.6)
Don't know	15	(4.7)
*Has she breastfed at least one child?*		
Yes	263	(81.7)
No	59	(18.3)
*Where did she give birth to her child(ren)?*		
China only	48	(15.0)
Ireland only	247	(77.2)
Both China and Ireland	25	(7.8)

^†^Columns where the numbers do not add up to 322 reflect missing values for this column.

^§^Mainland China (north) included the provinces of Anhui, Hebei, Heilongjiang, Henan, Hubei, Jiangsu, Jilin, Liaoning, Shandong, Shanxi; Xinjiang Uygur Autonomous Region; and the municipality of Beijing, Tianjin.

Mainland China (south) included the provinces of Fujian, Guangdong, Jiangxi, Sichuang, Yunnan, Zhejiang; Guangxi Zhuang Autonomous Region; and the municipality of Chongqing, Shanghai.

Hong Kong indicated Hong Kong Special Administrative Regions of China. In this study, one subject from Macau was also grouped into this category.

### Knowledge of breastfeeding

**Figure 1:**
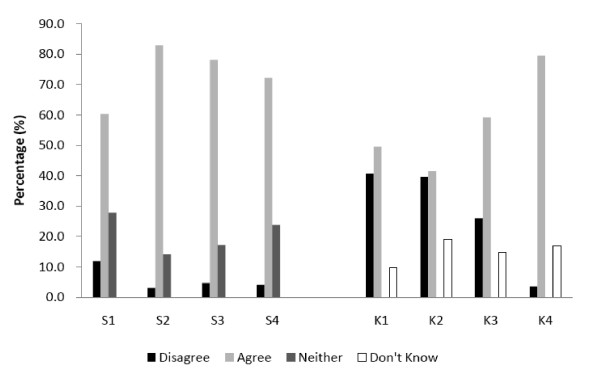
**Maternal knowledge of breastfeeding (n = 322)**. S1, S2, S3 and S4 are statements accessing the maternal awareness of the advantages breastfeeding for their babies. S1: A breastfed baby is likely to have fewer infections than a formula fed baby. S2: Breast milk is the ideal food for babies. S3: Breastfeeding provides health benefits for infants that cannot be provided by formula milk. S4: Breastfeeding significantly reduces the risk of a large number of infant diseases. K1, K2, K3 and K4 are misconceptions related to breastfeeding. K1.Infant formula should be fed to all newborn infants until their mothers' milk comes in. K2.The nutritional benefits of breast milk last only until the baby is weaned from breast milk. K3.Mother should not breastfeed if she catches a cold. K4.It is the frequency of breastfeeding as well as the milk removed from the breast that stimulates further milk production. Disagree = Strongly disagree/Tend to disagree; Agree = Strongly agree/Tend to agree

'Breastfeeding is better for the baby' was indicated as the main reason for breastfeeding (87%) (data not shown). According to Figure 1, more than 82% of them agreed that breast milk is the ideal food for babies (S2); over 70% were conscious of the unique health benefits of breast milk (S3) and above 60% recognized some disease protective effects of breast milk (S1&S4). Responses to these four awareness statements (S1-S4) was found to be highly reliable (Cronbach's alpha = 0.84). Mean score was above 4.0 (out of 5), implying that mothers were aware of the advantages of breastfeeding to the babies. Misconceptions were revealed according to another four statements (K1-K4) (Figure 1). Mothers were more likely to agree rather than disagree with these three statements: K1.Infant formula should be fed to all newborn infants until their mothers' milk comes in; K2.The nutritional benefits of breast milk last only until the baby is weaned from breast milk; and K3.Mother should not breastfeed if she catches a cold. And 17% were unsure about the stimulating effect of frequent sucking (K4).

### Attitudes towards breastfeeding

Maternal attitudes towards breastfeeding are outlined in Table 2. More than three quarters of the respondents denied that 'I do not like breastfeeding' and two-thirds agreed that formula-feeders missed one of the great joys of motherhood. Over 65% of mothers believed that breastfeeding was cheaper and would restore their figure more rapidly than formula feeding, but ambivalently over half of the respondents thought that breastfeeding would make mothers' breasts sag. The statements 'breastfeeding is old-fashioned' and 'formula feeding is a symbol of wealth' were highly disagreed with (> 90%). While mothers generally did not think they knew enough about breastfeeding (54.7%), their feeding choice would not be influenced by their husband/partner (77.4%) or infant formula advertising (78.3%). Attitudes less favourable to breastfeeding included: formula feeding was more convenient (53.6%) and a better choice for working/studying mothers (88%). Over 50% of the mothers would feel embarrassed to be seen breastfeeding and more than 65% would avoid breastfeeding in public. Over 85% of respondents considered certain traditional diets to be beneficial for breast milk production, and certain maternal foods having harmful effects (*e.g*. 'cold' foods according to Chinese food classification). A higher percentage of mothers believed that there were more breastfeeding promotion and education, as well as supportive public facilities in Ireland than in China. Living in Ireland was not deemed to be a barrier to breastfeeding.

**Table 2:** Maternal attitudes towards breastfeeding (n = 322)

	Disagree^†^	Agree^†^	Don't know
	(%)	(%)	(%)
I do not like breastfeeding.	78.7	17.1	4.1
Breast milk is less expensive than infant formula.	23.7	68.0	8.2
Breastfeeding can allow mother's weight to return to normal earlier than formula feeding.	12.3	65.2	22.5
Breastfeeding will make mothers' breasts sag.	30.0	53.6	16.4
Formula feeding is more convenient than breastfeeding.	42.0	53.6	4.4
Formula feeding is a better choice if the mother works or studies outside home.	9.1	88.0	2.8
Mothers who formula-feed miss one of the great joys of motherhood.	22.5	66.1	11.4
I would feel embarrassed if someone saw me breastfeeding.	45.7	51.7	2.5
I will not breastfeed in public.	31.6	65.2	3.2
Formula feeding is a symbol of wealth.	90.0	3.1	6.9
Breastfeeding is old fashioned.	96.6	0.9	2.5
I do not think I know enough about breastfeeding.	39.3	54.7	6.0
If husband/partner objects to breastfeeding, I will give up.	77.4	16.3	6.3
Infant formula advertisements have influenced my feeding decision.	78.3	17.0	4.7
Some traditional Chinese food can help to improve milk production.	5.3	85.2	9.4
Five classifications of foods (cold, sharp, hot, poisonous, windy) should be avoided during lactation because these foods have a negative effect on the nursing baby.	7.2	86.5	6.3
Public facilities (*e.g*. restaurants, shopping centre, public toilets) in Ireland are more supportive for breastfeeding practices than those in China.	8.1	82.2	9.7
There is more promotion and education on breastfeeding in Ireland than in China.	26.5	48.6	24.9
Living in Ireland is a barrier for Chinese women to breastfeed.	51.4	35.5	13.1

^†^Disagree = Strongly disagree/Tend to disagree; Agree = Strongly agree/Tend to agree.

### Socio-demographic influences on breastfeeding knowledge and attitudes

Univariate analyses demonstrated that mothers who were below 30 years old, less educated, both couples had non-professional occupations, and where the mother had not been breastfed as a baby were more likely to have lower awareness scores and agree with the misconceptions (Table 3). Less well-educated mothers, those from Hong Kong, and longer term Irish residents (> 10 years) had lower preferences for breastfeeding than their counterparts. The longer mothers lived in Ireland, the less likely they were to hold cultural belief concerning postpartum diets (Table 4).

**Table 3:** Mother's knowledge of breastfeeding, classified by socio-demographics characteristics‡ (n = 322)

	K1.Infant formula should be fed to all newborn infants until their mothers' milk comes in.				K3.Mother should not breastfeed if she catches a cold.				Maternal awareness of the advantages of breastfeeding for the babies.
	Disagree^†^	Agree^†^	Don't know	P	Disagree^†^	Agree^†^	Don't know	P	Mean score^§^
	**(%)**	**(%)**	**(%)**		**(%)**	**(%)**	**(%)**		

*Total*	40.7	49.5	9.8		26.0	59.2	14.7		4.03 ± 0.73
*Current age*									
20-29	34.9	54.7	10.4	0.013	20.8	70.8	8.5	0.005	3.90^b^
30-39	45.4	49.3	5.3		32.0	53.6	14.4		4.14^a^
> 40	38.9	40.7	20.4		20.4	53.7	25.9		4.03^ab^
*Education level*									
Primary/Secondary level	36.8	50.7	12.5	0.187	19.6	64.1	16.3	0.043	3.93^b^
Third level	44.2	48.5	7.3		31.9	54.8	13.3		4.14^a^
*Current occupation*									
Self-employed/Professional work	49.4	45.5	5.2	0.228	36.7	51.9	11.4	0.015	4.25^a^
Non-professional work	38.7	51.9	9.4		21.7	67.9	10.4		3.98^b^
Housewife	36.4	50.4	13.2		22.5	56.6	20.9		3.98^b^
*Husband/partner occupation*									
Self-employed/Professional work	51.9	39.6	8.5	0.013	36.4	50.5	13.1	0.011	4.20^a^
Non-professional work/Unemployed	34.7	55.4	9.9		20.7	64.0	15.3		3.97^b^
*Mother's birth place*									
Mainland China (north)	40.1	53.7	6.2	0.005	30.1	57.7	12.3	0.013	4.08^ns^
Mainland China (south)	40.2	50.5	9.3		22.2	65.7	12.0		4.04
Hong Kong	43.5	32.6	23.9		19.6	50.0	30.4		3.90
*Duration in Ireland (years)*									
< = 5	51.5	41.4	7.1	0.001	33.3	58.6	8.1	0.001	4.11^ns^
> 5-10	34.9	57.1	8.0		23.7	62.7	13.6		4.02
> 10	39.5	37.2	23.3		18.6	46.5	34.9		3.93
*Has mother herself been breastfed when she was a baby?*									
Yes	41.2	51.1	7.7	0.009	27.0	58.4	14.6	0.609	4.06^ns^
No/Don't know	37.8	40.0	22.2		20.0	64.4	15.6		3.91

^‡^Group comparisons for categories variables (variables in K1 and K2) were performed by Pearson Chi-square analysis. Group comparisons for continuous variables (mean score) were performed by One-way ANOVA analysis; ^ab ^different subscripts denote significances difference within groups using Scheffe *post hoc *multiple comparisons test (P < 0.05). ns: no significant difference (P > 0.05).

^†^Disagree = Strongly disagree/Tend to disagree; Agree = Strongly agree/Tend to agree.

^§^Mean score was calculated from responses basing on a 5-point Likert scale. Strongly Disagree = 1; Tend to disagree = 2; Neither = 3; Tend to Agree = 4; Strongly Agree = 5

**Table 4:** Maternal breastfeeding attitudes, classified by socio-demographic characteristics‡ (n = 322)

	I do not like breastfeeding.				Five classifications of foods should be avoided during lactation because these foods have a negative effect on the nursing baby.			
	Disagree^†^	Agree^†^	Don't know	P	Disagree^†^	Agree^†^	Don't know	P
	**(%)**	**(%)**	**(%)**		**(%)**	**(%)**	**(%)**	

*Current age*								
20-29	74.5	19.8	5.7	0.006	4.7	87.7	7.5	0.007
30-39	86.8	9.9	3.3		7.8	90.2	2.0	
> 40	65.4	30.8	3.8		9.4	75.5	15.1	
*Education level*								
Primary/Secondary level	72.2	22.5	5.3	0.024	5.2	88.2	6.5	0.413
Third level	84.8	12.2	3.0		9.1	84.8	6.1	
*Mother's birth place*								
Mainland China (north)	84.0	14.2	1.9	0.005	6.7	90.2	3.1	0.001
Mainland China (south)	77.4	15.1	7.5		4.7	88.8	6.5	
Hong Kong	62.2	33.3	4.4		15.2	67.4	17.4	
*Duration in Ireland (years)*								
< = 5	83.8	11.1	5.1	0.037	5.1	89.9	5.1	0.004
> 5-10	79.8	16.8	3.5		7.4	88.6	4.0	
> 10	62.8	32.6	4.7		11.6	69.8	18.6	

^‡ ^Group comparisons were performed by Pearson Chi-square analysis.

^†^Disagree = Strongly disagree/Tend to disagree; Agree = Strongly agree/Tend to agree.

### The association between of breastfeeding attitudes/knowledge and practices

Logistic regression analyses demonstrated that knowledge strongly independently associated with practices (Table 5). Mothers who had higher awareness scores (OR = 2.98, CI: 1.87-4.73), or who were opposed to feeding newborns with formula at birth (OR = 2.96, CI: 1.40-6.27), or who believed in the stimulation of breast milk production by frequent sucking (OR = 3.49, CI: 1.76-6.92), or who did not see the need to stop breastfeeding if the mother caught a cold (OR = 3.14, CI: 1.16-8.49) were about three times more likely than their peers to breastfeed. As for attitudes, disagreement with the statement 'I do not like breastfeeding' was the strongest determinant of breastfeeding (OR = 4.98, CI: 2.50-9.90). Mothers who believed in the positive effect of the traditional Chinese postpartum diet on breast milk production (OR = 3.82, CI: 1.70-8.60), or who agreed that 'mothers who formula-feed miss one of the great joys of motherhood' (OR = 3.17, CI: 1.64-6.13), or who disagreed that 'breastfeeding will make mothers' breasts sag' (OR = 2.28, CI: 1.01-5.12) or who denied that 'if husband/partner objects to breastfeeding, I will give up' (OR = 2.31; CI: 1.17-4.52) were more likely to breastfeed (Table 5).

**Table 5:** Knowledge and attitudinal factors influencing breastfeeding practice, performed by binary logistic regression analysis§ (n = 322)

Items	P	Odds Ratio (95% Confidence Interval)
Awareness (score) of the advantage of breastfeeding.	< 0.001	2.98 (1.87-4.73)
		
Infant formula should be fed to all newborn infants until their mothers' milk comes in.^(D)^	0.005	2.96 (1.40-6.27)
		
It is the frequency of breastfeeding as well as the milk removed from the breast that stimulates further milk production. ^(A)^	< 0.001	3.49 (1.76-6.92)
		
Mother should not breastfeed if she catches a cold. ^(D)^	0.024	3.14 (1.16-8.49)
		
I do not like breastfeeding. ^(D)^	< 0.001	4.98 (2.50-9.90)
		
Breastfeeding will make mothers' breasts sag.^(D)^	0.046	2.28 (1.01-5.12)
		
Mothers who formula-feed miss one of the great joys of motherhood. ^(A)^	0.001	3.17 (1.64-6.13)
		
If husband/partner objects to breastfeeding, I will give up. ^(D)^	0.015	2.31 (1.17-4.52)
		
Some traditional Chinese food can help to improve milk production. ^(A)^	0.001	3.82 (1.70-8.60)

^§^Socio-demographic variables including maternal current age, maternal birthplace, mother's duration in Ireland (continuous variable), education level, whether or not mother herself had been breastfed when she was a baby, and husband/partner's occupation, were adjusted. Only one attitude item was examined each time. This table only present the items which were found to be significant in the model.

^(A) ^Reference subgroup: Combination of 'Disagree' and 'Don't know'.

^(D) ^Reference subgroup: Combination of 'Agree' and 'Don't know'

## Discussion

### The sample

According to the Irish census in 2006, there were 4660 Chinese females between 20 and 54 years old living in Ireland [18]. Of these, 322 participated in this study, representing 9% of the Chinese women in the age group in Ireland. Since there are no census data specifically on Chinese mothers, it is difficult to determine the generalisability of the results. Comparing to the present sample profile to the census data on Chinese women in Ireland [18], higher percentages of married (85% *vs*. 65%), tertiary level educated (51.6% *vs*. 28%), and working (58.7% *vs*. 38%) women were found in this study population. Participants were generally in a relatively low social class, which was reflected by their low income and couple's non-professitional occupation. This is in accordance to the census data that the majority of Chinese in Ireland are students or in non-professional work [18]. The majority of participants had ever breastfed and had been breastfed as a baby; this reflects the strong Chinese breastfeeding culture. Most participants (77.2%) had their first babies after coming to Ireland.

### Breastfeeding knowledge

Chinese mothers in this study were by and large aware of the advantages of breastfeeding for babies, in agreement with the Chinese migration study in Australia conducted by Li *et al*. [16]. However, misconceptions revealed in the present study were less prevalent among the Chinese mothers in Australia [16]. In the present study, mothers agreed to feed the newborn with formula before breast milk came in, contradicting to the WHO infant feeding recommendations [31]. Thus health professionals in Ireland need to encourage attachment to the breast as soon as the baby is born. Mothers in this study were not aware of the long term health benefits of breastfeeding, suggesting a need to emphasize this information in breastfeeding education in Ireland, and some scientific evidence might be given by way of example. In addition, many mothers thought they should discontinue breastfeeding if they caught a cold, however, medical advice indicates that continued breastfeeding has a protective effect for the baby if the mother has already suffered from a cold [32]. Breastfeeding education should inform mothers more on the medical situations when breastfeeding should or should not be advised [33].

### Attitudes towards breastfeeding

Attitudes favourable to breastfeeding found in this study, including a preference for breastfeeding, the belief that 'formula-feeders miss one of the great joys of motherhood' and 'breast milk is cheaper than infant formula', have also been reported among Chinese mothers in Australia, where an even stronger preference (91%) for breastfeeding exists [16]. These positive attitudes might be attributed to the Chinese breastfeeding culture [16,34,35]. Although mothers believed that breastfeeding would help them to regain their figure faster than formula feeding, they also agreed that breastfeeding would make mothers' breasts sag. Ambivalent outcomes were also reported by Li *et al*. [16] in Australia, but not by Kong & Lee [30] in Hong Kong, where far fewer perceived this negative effect to the shape of breasts [30].

Breastfeeding was considered to be inconvenient among Chinese mothers in Ireland. This result was contrasted with the finding of Li *et al*. [16] in Australia but was consistent with the findings of some other migration studies in Australia and Europe [10,13,14]. In an attempt to solve the inconvenience of breastfeeding, mothers could be encouraged to use breast pumps, which allow other caretaker to participate in infant feeding by using breast milk rather than formula in the bottle. Embarrassment and the unwillingness to breastfeed in public were demonstrated among Chinese mothers in Ireland. Culture may account for this. The Chinese have always regarded the breasts as sexual objects. This has affected them to the extent that not many breastfeed in public [29]. This attitude is not unique to the Chinese culture. Studies in Ireland have also reported very strong negative response to breastfeeding in the public and cultural values which in turn have made formula feeding the norm [24,27,36,37]. However, in Australia where breastfeeding is predominant and people generally breastfeed in public, 89% of Chinese immigrant mothers denied that breastfeeding was too embarrassing, and 82% did not mind breastfeeding in front of others [16]. Accordingly, in spite of the cultural influence, environmental influence seemed to weight more on the issue of breastfeeding in public. Therefore, breastfeeding campaigns in Ireland need to increase the acceptance of breastfeeding in public as well as facilitate a more breastfeeding friendly environment *via *providing appropriate public facilities, and giving mothers information on clothing and positioning techniques to minimize exposure of the nursing mother. The belief that 'infant formula feeding is a better choice for a working mother' may be on one hand associated with the perceptions of breastfeeding being inconvenient and embarrassing in this study. On the other hand, the workplace environment not being conducive to breastfeeding may account for this [38,39]. Health sectors should evaluate the working environment when establishing strategies to improve breastfeeding in Ireland.

In this study, husband's attitudes and infant formula advertisements were not considered to be a strong influence on the maternal infant feeding decision. This may be explained by the Chinese culture that women are mainly supported, guided and looked after by their mothers or female relatives, and women's breastfeeding practices are positively associated with the information and support they receive from their mother or mother-in-law. The main role for a husband is considered to be to provide for the financial needs of the family [40]. A study in Hong Kong indicated that only 9.6% mothers ranked husband's advice as the most important factors determining their infant feeding decision [30]. At the same time, a lack of breastfeeding knowledge was perceived among themselves by the Chinese mothers in Ireland, this may be due to less information and support being received from their mothers far away in China.

Cultural beliefs in the positive effect of a traditional postpartum diet on breast milk production predominated in this survey population. For those who consume the 'special Chinese diet', this cultural belief may help to remove the commonly perceived barrier, insufficient breast milk [15,41], and in turn psychologically help to prolong the breastfeeding duration. On the contrary, for those who are not able to consume the 'special diet', this belief may have some negative influence to their breastfeeding duration. There is also some evidence among Vietnamese immigrants to support the importance of confinement foods to lactation [40,42]. Therefore, the availability and accessibility of traditional Chinese foods in Ireland may be essential to optimize breastfeeding duration. The longer mothers lived in Ireland, the less likely they were to hold the cultural belief in the efficacy of traditional postpartum diets. This is suggestive of the intra-individual shifts over time in the beliefs of immigrant mothers.

Chinese mothers in this study had more misconceptions and negative attitudes towards breastfeeding than Chinese mothers in Perth, Australia [16]. Variations in socio-demographic status may account for this difference. Chinese mothers in the present study were in a relatively lower socio-demographic status than the participants in Perth, Australia, which was reflected by the lower percentages of maternal third level educational attainment (51.6% *vs*. 76.3%), mother having professional work or self-employed (24.9% *vs*. 38%), and husband having professional work or self-employed (34.8% *vs*. 80.9%). On the other hand, the influence of the host environment may also account for the gap in breastfeeding knowledge and attitudes between these two groups of migrant Chinese mothers. Australia has a series of breastfeeding programmes, including those specifically talored to ethnic groups [43]. If there was as much education and promotion specific to ethnic groups in Ireland as in Australia, and if breastfeeding was as prevalent and acceptable in Ireland as in Australia, the gap would perhaps diminish.

### The association between of breastfeeding attitudes/knowledge and practices

Maternal breastfeeding knowledge and attitudes were found to be influential to infant feeding behaviour, corroborating other studies [22,23,27]. Those who reported that they liked breastfeeding and enjoyed doing so were also more likely to breastfeed. They had indicated a strong willingness to breastfeed and that they were not easily influenced by others. This suggests that in promoting breastfeeding, it may be more effective to publicize it as enjoyable rather than a maternal responsibility, and target at those who have lower self-efficacy of breastfeeding. Culture belief should not be ignored, as who believed in the efficacy of traditional Chinese diet were more likely to breastfeed. More research into the cultural influence would facilitate the provision of effective, consistent, and culturally sensitive education and intervention.

### Socio-demographic influences on breastfeeding knowledge and attitudes

In this study, those having inaccurate breastfeeding knowledge were more likely to be younger (< 30 years old), less educated and less affluent mothers, as well as having not herself been breastfed as a baby. Less educated mothers also had less positive attitudes towards breastfeeding. These findings can be explained by the recent literature on the relationships of maternal age [44], education [45], economic status [46], mother herself having been breastfed as a baby or not [47] and breastfeeding practices. Length of stay in Ireland was found to be inversely associated with breastfeeding knowledge and attitudes. This was similar to the Chinese study in Australia, in which the authors attributed this to social isolation and difficulties in communicating in English [16]. However those reasons might not be generalized to our study, because breastfeeding in Ireland is much less prevalent than that in Australia. The longer staying Irish residents becoming less knowledgeable about and positive towards breastfeeding was probably due to acculturation, *i*.*e*. adapting themselves to the formula feeding culture of Ireland. Hong Kong mothers were found to have less knowledge, less positive attitudes and weaker cultural beliefs of breastfeeding than mothers from mainland China. This can be explained by the fact that breastfeeding rates in Hong Kong (less than 67%) are lower than that in mainland China (about 80%) [9,39,48-51]. However, previous migration studies did not specifically separate Chinese mothers from Hong Kong and from the mainland [13,14,16], thus the attitudinal differences revealed in this survey may need to be considered and verified in future studies.

### Limitation

The generalisability of the study findings is limited due to the use of convenience sampling. Any conclusions that were reached may be applicable only to people who share the characteristic of this sample. However, since the contact information of Chinese mothers in Ireland is unavailable, and considering the study constraints of time and cost, the strategy of convenience sampling is more feasible than probability sampling in this study. Mothers with third level education dominating in the study participants may be a source of bias. Further study among less well-educated Chinese mothers in Ireland is warranted. Due to the snowball method in data collection, it was not possible to obtain a response rate in this study. This problem however has been a feature in other migrations studies [40,52]. The inflation of alpha-error in one way ANOVA analysis and multiple comparisons should also be acknowledged. In spite of these limitations, a variety of strategies have been used to ensure the validity and reliability of this study, including Mandarin and Cantonese-speaking researcher, and translation followed by 'blind' back translation of the questionnaire.

## Conclusions

This study explored the maternal breastfeeding knowledge, attitudes and cultural beliefs of Chinese mothers who are currently living in Ireland. In spite of the general awareness of the advantages of breastfeeding, misconceptions, negative attitudes and strong cultural belief found in this study suggest a need to emphasize the long-term benefits of exclusive breastfeeding for the first six months, to improve the social acceptance of breastfeeding in public, and to offer culturally appropriate support to Chinese mothers in Ireland. Maternal breastfeeding knowledge and attitudes were strongly associated with breastfeeding practice in this study, those who were less knowledgeable (younger, less well-educated, in a lower social class and had not been breastfed as a baby) and less inclined to breastfeed (less well-educated, Hong Kong mothers, and longer staying Irish residents) should be targeted for breastfeeding education and intervention.

## Competing interests

The authors declare that they have no competing interests.

## Authors' contributions

QZ, KMY and JMK were responsible for the study design and the interpretation of the results (JMK was the study coordinator/supervisor). QZ was responsible for data collection, input and analysis, as well as the write-up of the draft manuscript. KMY and JMK contributed to the editing of the final manuscript. All authors read and approved the final manuscript.

## Pre-publication history

The pre-publication history for this paper can be accessed here:

http://www.biomedcentral.com/1471-2458/10/722/prepub
